# Possible Therapeutic Potential of Disulfiram for Multiple Myeloma

**DOI:** 10.3390/curroncol28030193

**Published:** 2021-06-03

**Authors:** Denisa Weiser Drozdkova, Katerina Smesny Trtkova

**Affiliations:** 1 Department of Clinical and Molecular Pathology, Faculty of Medicine and Dentistry, Palacky University, 77900 Olomouc, Czech Republic; katerina.smesny@upol.cz; 2 Institute of Molecular and Translational Medicine, Faculty of Medicine and Dentistry, Palacky University, 77900 Olomouc, Czech Republic

**Keywords:** multiple myeloma, disulfiram, relapses, pharmacoresistant, therapy

## Abstract

Multiple myeloma (MM) is a malignant disease of the plasma cells representing approximately 10% of all hemato-oncological diseases. Detection of the disease is most probable at around 65 years of age, and the average survival of patients is estimated to be 5–10 years, specifically due to frequent relapses and resistance to the therapy used. Thus, the search for new therapeutic approaches is becoming a big challenge. Disulfiram (DSF), a substance primarily known as a medication against alcoholism, has often been mentioned in recent years in relation to cancer treatment for its secondary anti-cancer effects. Recent studies performed on myeloma cell lines confirm high inhibition of the cell growth activity if a complex of disulfiram and copper is used. Its significant potential is now being seen in the cure of haematological malignities.

## 1. Characteristic of Multiple Myeloma

Multiple myeloma is a disease of terminally differentiated B lymphocytes with the subsequent formation of malignant plasma cells, excessively producing monoclonal immunoglobulin, the so called M-protein [1]. Multiple myeloma affects men more often than women and is up to two times more prevalent in African Americans [2]. Before the actual development of multiple myeloma, patients undergo two stages. The first precursor stage is monoclonal gammopathy of undetermined significance (MGUS), accompanied by a low blood level of the M-protein as well as the number of clonal plasma cells in the bone marrow being under 10%, where the risk of transition from MGUS to active myeloma is only 1% [3]. The next stage of the disease is called smouldering myeloma (SMM), when a higher blood level of the M-protein can be detected and the number of clonal plasma cells is rising (10–20%). Overall, the risk of progression into an active myeloma is already 10% [4,5,6]. Active myeloma is represented by typical clinical symptoms, and the following three criteria must be met for its diagnosis: number of plasma cells in bone marrow (BM) is >10%, M-protein is present in the blood and/or urine, at least one organ dysfunction or damage is present—the CRAB criteria (Table 1) [7]. 

Among the main symptoms are gradual destruction of the bones, which is manifested by back pain, including night pain at rest and osteoporosis leading to subsequent pathologic fractures. Due to impaired bone marrow function, the patients suffer from fatigue and weakness. Infectious diseases occur more frequently due to the suppressed immunity. A higher concentration of the M-protein causes increased viscosity of the blood, which leads to kidney damage and subsequently to renal failure. Neurological symptoms are not uncommon either and can have the character of peripheral neuropathy [6,9].

The diagnosis of multiple myeloma includes blood count, serum and urine protein analysis, erythrocyte sedimentation rate, and imaging (X-ray, MRI). When suspecting the disease, we perform a biopsy of the bone marrow and consequent morphological assessment of the bone marrow smear. The signs of malignant character include the presence of nuclear inclusions (so called Dutcher bodies) and multinucleated cells. Another important indicator for stating the diagnosis of MM is the assessment of individual light chain concentrations as well as determination of the abnormality of their ratio. M-protein typically belongs to the IgG class (about 50% of all MM). MM related to secretion of paraprotein from the IgM class is often accompanied by hyperviscosity of the serum. Sometimes, the paraprotein consists only of light chains of immunoglobulin [10].

In recent years, there has been extraordinary progress in the treatment of symptomatic multiple myeloma, particularly with the arrival of proteasome inhibitors (such as bortezomib) and immunomodulation agents (such as lenalidomide) [11]. The treatment starts with chemotherapy, and the main active substances are bortezomib and dexamethasone. In most cases, however, these preparations are combined with cyclophosphamide or adriamycin for better efficiency [10]. This combination is often replaced by bortezomib with lenalidomide, which shows better results, with the same level of toxicity [11]. Younger patients or patients with high risk (17p deletion, extramedullary disease) undergo combined autologous/allogeneic stem cell transplantation [10,11]. Because there is no curative treatment, MM inevitably relapses. The subsequent relapses then occur with increasing frequency and become more and more pharmacoresistant [11]. Concepts for improving or maintaining remission are being researched in an effort to delay the relapse of multiple myeloma. Cytostatic drugs, steroids, and interferon, as well as thalidomide, have been tested; however, most have been abandoned due to significant adverse effects and insufficient efficiency in some cases [10].

### 1.1. Development of B-Lymphocytes and Plasma Cells Is Crucial for the Pathogenesis

It is supposed that the presence of somatic mutations in immunoglobulin genes, without subsequent remodeling, is the origin of MM pathogenesis. These mutations occur during the development of B-lymphocyte, an immune system cell, which is responsible for a specific humoral antibody response. B-lymphocytes are generated from haematopoietic stem cells based on the transcription signals of the PU.1, E2A, and PAX5 genes. B-lymphocytes feature a BCR (B-cell receptor) on their surface, which is produced in the stage of the pro-B cell thanks to the activation of the RAG gene and after launching a process known as VDJ recombination [12]. The B-cell receptor develops during the pro- and pre-B lymphocyte stages up to the stage of immature B-lymphocytes, where the BCR is fully mature and the cells express IgM on the surface. In this developmental phase, elimination of the autoreactive clones occurs. The immature B-lymphocytes migrate as transitional B-lymphocytes, expressing the surface IgM and IgD, to the secondary lymphatic organs, where they divide into follicular B-lymphocytes and marginal zone lymphocytes [13,14]. Here they wait in the neighborhood of the follicular T-helper lymphocytes for reaction with an antigen. If they do not meet with the antigen, they remain in the G0 stage [15].

After activation by the native antigen, the mature naïve B-lymphocyte produces short-term plasma cells. Additionally, activation of B-lymphocytes in germinal centers occurs, together with activation of follicular T-lymphocytes. This cascade of processes is accompanied by several temporary stages called the germinal center reaction. Among these temporary stages belong clonal expansion, class-switch recombination (CSR) on the IgH locus, somatic hypermutation, and affinity maturation of VH sequences, which allow for antigen recognition. Somatic hypermutation and class-switch recombination are initiated by the *Activation-Induced Cytidine Deaminase* (AID) enzyme. AID is aimed at single-stranded DNA and deaminates deoxycytidines (dC) in DNA to deoxyuridine (dU) during the G1 phase of the cell cycle. The activity of AID and processing of the resulting deoxyuridine susceptible to faulty transcription increases the speed of mutations in the immunoglobulin genes by an estimated six orders of magnitude (~10^–9^ to ~10^–3^ mutations per one base pair per division) [16]. Beside the immunoglobulin gene recombination in the bone marrow, these temporary stages represent the second step of diversification [14,15,17]. Consequently, division to memory B-lymphocytes and plasmablasts occurs and they represent the final stage of the mutation process. These cells migrate to the bone marrow, where, thanks to the stromal cells, the terminal differentiation into no longer dividing plasma cells takes place, and these continue to survive in the bone marrow for months to years [18,19,20]. Thanks to somatic hypermutation, the memory B-lymphocytes and plasma cells increase affinity to the antigen and change the immunoglobulin (Ig) isotypes, thus expressing subsequent Ig isotypes (IgG, A, or E). Some plasma cells and memory B-lymphocytes, however, can continue IgM expression [21].

The development of long- as well as short-term plasma cells depends on the expression of Blimp-1 protein, coded by the PRDM1 gene [12]. Blimp-1 is found in all plasma cells, including those that are created during the primary and secondary reactions from memory cells and in long-term plasma cells in the bone marrow [22]. Blimp-1 is the main regulator of plasma cell differentiation as it directly suppresses the transcription factors by activation of the C-MYC, BCL6, and PAX5 genes [12,23]. Thus, Blimp-1 induces the plasmacytic differentiation and inhibits the alternative development of mature B-lymphocytes [23].

Long-term plasma cells are non-dividing terminally differentiated cells. They demonstrate high secretion of the Ig antibodies, but also the expression of surface markers such as CD38, CD19, and Syndecan-1 [24]. The viability of the cells is influenced by microenvironment of the bone marrow. The key factors for long-term survival of the plasma cells are IL-21, BAFF, and APRIL from the TNF family [24,25,26]. On the plasma cell surface, the BCMA receptor is highly expressed, on to which BAFF and APRIL are binding. Thanks to the initiation of the BCMA receptor, the activation of the NF-κB pathway occur, increasing antiapoptotic protein Mcl-1 gene expression, which is crucial for the long-term survival of the plasma cells [24,26]. IL-21, as well as IL-6 and IL-10, initiate the activation of STAT3, which is important for interaction of the plasma cells with different types of cytokines. Plasma cells play a key role in maintaining lifelong humoral immunity, and their long-term presence is necessary for this function. Therefore, discerning the molecular mechanisms allowing their long-term survival is a subject of current research [26].

### 1.2. Role of Plasma Cells in Disease Progression

Generating high-affinity antibodies specific for the antigen is crucial for the organism’s immunity reaction to the antigenic challenge. Multiple myeloma undergoes malignant reversal in the stage of the clonal plasma cell, causing production of the monoclonal paraprotein (M-protein); so far, no trigger mechanism is known [27,28]. The reversal consists of pathological cascade of events influenced by accumulation of the cytogenetic changes in the cell (plasmacyte) as well as epigenetic factors, resulting likewise in dysregulation of the cell cycle [29].

Genetic changes that occur during the progression of the disease can be divided into primary and secondary. Primary events further divide into hyperdiploid (HRD) and non-HRD subtypes, which are defined by a row of repetitive chromosomal translocations. Primary HRD are usually triple odd numbers on chromosomes 3, 5, 7, 9, 11, 15, 19, and/or 21 [30,31]. Primary non-HRD events include translocations of the immunoglobulin heavy chains (IgH), the most frequent translocations being t (11; 14), t (4; 14), and t (14; 16). In addition to this, del13q is the most common deletion in MM [29]. Translocation t (11; 14) relates to high expression of Bcl-2 and low expression of Mcl-1/Bcl-XL [32]. Translocation t (14; 32) leads to the juxtaposition of the nonimmunoglobulin loci in DNA sequences and specifically activates the oncogenes. Translocations of these loci are detected in up to 75% of MM cases, which leads to the hypothesis that chromosome translocations can cause the initial transformation of a plasma cell to a malignant cell [33].

An example of chromosomal translocation is translocation of the gene for cyclin D1 at the 11q13 locus with the consequence of an excessive expression of cyclin D1 [34]. The inhibitor of cyclin-dependent kinase p27Kip1 is bound by cyclin D1, blocking the inhibitor and allowing progression of the cell cycle to the G1 phase. The result is tumorigenesis in myeloma cells. Other significant genetic mutations contributing to the development of multiple myeloma are NRAS and KRAS, along with excessive expression of the c-Myc protein [33].

In the pathogenesis of MM, development of the malignant clone is influenced by genetic change; however, the interaction between malignant plasma cells and the microenvironment of the bone marrow is of the same importance for the survival and further progression of myeloma cells. One of the main cytokines, produced in bone marrow, which promotes the progression and growth of the malignant plasma cells is interleukin-6. The main source of IL-6 are stromal cells of the bone marrow, macrophages, osteoblasts, osteoclasts, and even some myeloma cells [35]. IL-6 is a pro-inflammatory cytokine that supports the signaling pathways of NF-κB, MAPK, and PI3K/Akt. Together, these pathways support anti-apoptotic signaling and resistance to drugs in cancer cells [36]. Although some myeloma cells produce IL-6, the main source is the stromal cells of the bone marrow, as well as macrophages, osteoblasts, and osteoclasts [35].

Expression of the growth factors IL-6 or BAFF in MM is regulated by the pathway NF-κB as well as the expression of regulators of the cell cycle—cyclin D, cyclin E, c-Myc, and E2F3α [37]. NF-κB signaling, in combination with other important transcription factors, such as STAT3, also plays an important role in the regulation of apoptosis [38]. Mutations of NF-κB, typically generated in the MM tumors, mostly activate the non-canonical pathway (NIK, TRAF3, TRAF2, CD40, etc.), while the canonical pathway is also influenced (CYLD, NF-κB1, TACI, etc.); however, usually both pathways are influenced [37]. Thus, NF-κB modulates adhesion molecules on the tumor cell and in the microenvironment, and as a transcription factor it mediates the survival of cells and drug resistance in multiple myeloma [39].

Malignant plasma cells produce large amounts of monoclonal paraprotein and feature expansive and highly developed endoplasmic reticulum [40,41]. Chemical influences or nutrient deprivation can impair the protein formation in the endoplasmic reticulum (ER), causing stress of the ER and thus activation of the signaling pathway unfolded protein response (UPR). UPR is a complex multimolecular apparatus monitoring ER stress types and subsequently launching several signaling pathways. The result of UPR activation is lowered protein synthesis and increased transcription of the ER resident chaperones and other components of the protein degradation apparatus, to prevent accumulation of misfolded proteins. Activation of the UPR pathway and the cell survival pathway is launched in parallel, leading to the activation of AKT, ERK, and IAP, which antagonize apoptosis and provide the UPR system time for repairs. However, if the stress is intense or long-term, the activation of UPR eventually leads to cell cycle arrest and apoptosis induction [28,41,42]. Apoptosis is realized through activation of the C/EBP homologous protein (CHOP), with consequent suppression of the BCL-2 transcription and cell cycle arrest. Next is activation of the intrinsic apoptotic pathway by direct caspase-4 activation, releasing the cytochrome c from mitochondria that subsequently bonds to Apaf-1 and caspase-9, leading to caspase-3 activation and apoptosis [28].

The second mechanism participating in the adaptation to the endoplasmic reticulum stress is autophagy. Plasma cells show higher levels of basal autophagy compared to other tumors, whereas autophagy is essential for MM cells survival [7]. The primary function of autophagy is to maintain cell metabolism under the conditions of cell starving. Because the plasma cells produce antigens, autophagy plays a key role in the processing and elimination of misfolded immunoglobulins. Typically, autophagy is induced after inhibition of the mTOR protein kinase, which is the key regulator of cell growth. Additionally, nuclear protein p53 can trigger the autophagy by increasing the AMPK or DAPK activity, with subsequent phosphorylation of the Beclin-1 protein. However, compared to nuclear p53, the cytoplasmic protein p53, on the contrary, can activate mTOR and inhibit the autophagy [7,26].

## 2. Disulfiram

Disulfiram (DSF), a substance primarily known as a drug against alcoholism, has been frequently mentioned in recent years in relation to cancer therapy for its secondary anti-tumor effects [43]. A significant potential is seen in the therapy of hematologic malignancies, mainly in the treatment of multiple myeloma (MM).

Disulfiram was discovered in 1881 by a German chemist and was later used in the textile and rubber industry. Only in 1947 did it start to be tested in Denmark as an antiparasitic, and due to the adverse reactions to concurrent use of alcohol, disulfiram was approved by the FDA in 1951 as a drug against alcoholism and is today known as Antabus [44]. In 2017, Skrott published the relationship of deaths associated with cancer in patients treated for alcoholism by DSF and subjects who did not use DSF. He found that the cancer patients had a lower risk of death when using DSF. This finding was an impulse for further research of this substance as an anti-cancer agent [45].

Disulfiram has been used to treat alcohol dependence for 70 years and its effects on the body are well described. Disulfiram is absorbed slowly from the gastrointestinal tract (80 to 90% of oral dose). However, a 12 h period is required for its full action. Disulfiram excretion from the body is relatively slow, and at the end of the week about 1/5 of the dose remains in the body. Its biotransformation is predominantly hepatic. Most of the absorbed drug is excreted in the urine as sulphate, partially free and partially esterified [46,47]. With chronic disulfiram dosing, carbon disulphide may also be excreted by the lungs. This results in side effects such as a metallic taste and bad breath. The side effect profile further includes fatigue, dermatitis, impotence, peripheral neuropathy, liver damage, and changes in mental status, including psychosis. However, two placebo-controlled studies did not show a higher incidence of adverse events with 250 mg/daily [46,48].

Disulfiram (tetraethylthiuram disulfide) is a quaternary amino compound with the molecular formula C_10_H_20_N_2_S_4_ [49]. In the body, DSF is metabolized to deithyldithiocarbamate (DDTC), and by a gradual transfer and oxidation catalyzed by P450, metabolites are created, which are directly involved in the inhibition of the aldehyde dehydrogenase (ALDH) enzyme [50]. Under standard conditions, ALDH transforms alcohol in the liver and brain to acetaldehyde and oxidizes a side product of acetaldehyde to acetic acid. When the ALDH enzyme is inhibited, higher levels of acetaldehyde in blood occur, leading to so called disulfiram-alcohol reaction (DER) [46].

**Figure 1:**
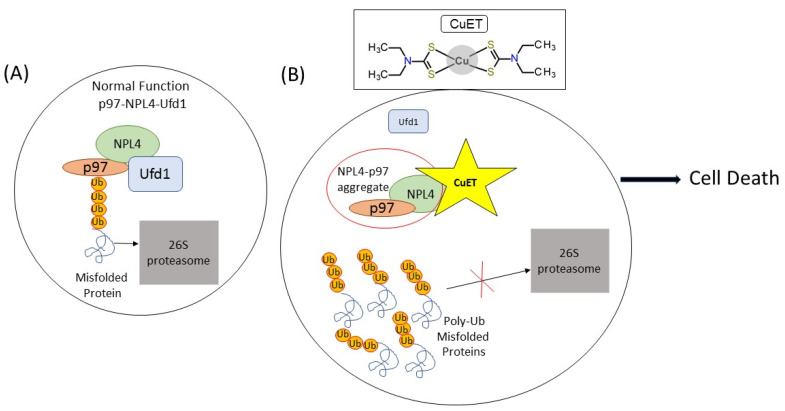
Simplified scheme of the CuET action. (**A**) Under normal condition, NPL4 and Ufd1 cofactors bind p97/VCP segregase. The p97-NPL4-Ufd1 complex allows the processing of ubiquitinated proteins with the 26S proteasome. (**B**) The CuET binds to NPL4 and causes aggregation of p97-NPL4. Therefore, the p97-NPL4-Ufd1 complex is not formed; ubiquitinated proteins accumulate in the cell, leading to cell death.

DDTC, which is not further metabolized, forms a complex with copper ions creating bis (diethyldithiocarbamate)—CuET [51]. This metabolite is the main compound with anti-tumor potential. CuET causes aggregation and dysfunction of NPL4, an essential cofactor of the p97/VCP segregase, thereby deactivating the p97-NPL4-UFD1 pathway (Figure 1). This protein complex is important for many cell processes, including the pathway called endoplasmic-reticulum-associated degradation (ERAD). Thanks to ERAD, the misfolded or short-term polypeptides from the endoplasmic reticulum and are conjugated with ubiquitin and further degraded by 26S proteasome [45,52].

### Disulfiram Treatment Potential

Nowadays, the most frequently used drugs for the treatment of MM are still the proteasome inhibitors (bortezomib, carfilzomib, and ixazomib) [38]. The suppression of the proteasome function blocks the ERAD mechanism, leading to accumulation of misfolded proteins. This induces stress of the endoplasmic reticulum, which affects the MM cells. If this state lasts longer, it can lead to cell cycle arrest and apoptosis induction [37]. Moreover, disulfiram offers the potential of a similar mechanism of protein degradation inhibition because, as mentioned above, the CuET complex binding NPL4 cofactor inhibits NPL4, which is involved in the p97-dependent processes, leading to protein degradation [53].

Bortezomib is a reversible 26S proteasome subunit inhibitor; at the same time it has an inhibitory effect on the NF-κB pathway. Because IκB is a substrate of proteasome and its degradation is inevitable, the inhibition of proteasome by bortezomib can lead to increased cytoplasmic levels of IκB, resulting in negative regulation of the NF-κB target genes [37,54,55]. Another important fact is that NF-κB has been identified as a mediator of paracrine signaling between multiple myeloma cells and bone marrow stromal cells (BMSC). The activation of IL-6 in the bone marrow dependent on NF-κB is induced by the adhesion to the MM cells or their secretion of TNF-α. Bortezomib blocks the activation of the NF-κB induced by TNF-α, leading to reduction of relations between multiple myeloma cells and bone marrow stromal cells and related decrease in secretion of IL-6, which supports their survival [55].

Unfortunately, the prognosis of myeloma patients whose disease is no longer reacting to the proteasome inhibitors remains miserable. Therefore, innovative therapies with different mechanisms of action are required [32]. Current medications are often combined; thus they are aimed at a diverse spectrum of molecular pathways. Beside the drugs targeted at specific MM mechanisms, drugs disturbing the synthesis of DNA and RNA, supporting cell apoptosis and inhibiting angiogenesis, are often used in the treatment [38].

Specific regimens for relapsing and refractory MM disease are based on the available substances, and the patients are recommended to participate in clinical studies. One of these studies is specifically based on the application of disulfiram. It is an open-label phase-I study, initiated in February 2021 at the University of Utah. In this study, disulfiram is used along with copper gluconate (NCT04521335). Studies performed on myeloma cell lines show high inhibition of cell growth activity when using disulfiram with copper.

After influencing three myeloma cell lines and patient samples, followed by evaluation of MM cell growth inhibition, the growth of cells influenced by DSF with Cu was noticeably reduced in a time and concentration dependent manner. Antiproliferative IC50 was 0.5 μM for the combination of DSF and copper, which is a lower concentration than administered to patients treated for alcohol addiction. Besides, the treatment by DSF and Cu is associated with the reduction of Akt and MAPK /Erk expression and with activation of phospho-p38 MAPK. Activated p38/MAPK phosphorylates Bcl-2 in the mitochondrial compartment leads to cytochrome c release, activation of caspases, and apoptosis. Simultaneously, a dysregulation of cell redox state, ROS generation, and change of the mitochondrial membrane potential has been observed when using disulfiram with copper, which can also initiate mitochondrial apoptotic pathway activation [56]. Equally, the effect of ROS on arresting cell cycle in the G1 phase and induction of apoptosis has been described in the cells of acute myeloid leukaemia and glioblastoma after being influenced by DSF with Cu [57,58].

Mechanisms of action of DSF with Cu leading to apoptosis have been described in the cells of myeloma lines as well as plasma cells isolated from the bone marrow of MM patients. In the myeloma line, cell cycle arrest in the G2 phase occurred. DSF with Cu dose-dependent cell apoptosis has been detected by the FACS method. Upon inhibition of apoptosis of the MM cells by the zVAD substance, the dependence and independence of cell apoptosis on caspases has been proved. Apoptosis, induced by the combination of DSF and Cu, has also been observed in the cells isolated from patients with diagnosed relapsing/refractory MM. To determine the role of the intrinsic apoptotic pathway in the dose of DSF with Cu leading to apoptosis, the mitochondrial transmembrane potential (MMP) in the influenced MM cells was assessed. The treatment led to the loss of MMP in myeloma cells, which can be an indicator of mitochondrial membrane permeability and induction of the intrinsic apoptotic apparatus [59].

The treatment by the complex of DSF with Cu increases the activation of caspase-8 and caspase-3 and the accumulation of PARP cleavage fragment, which confirms, on the protein level, their involvement in the signaling pathways of cell apoptosis. Beside this, an increased phosphorylation of JNK and c-Jun has been detected, leading to the induction of apoptosis. Influencing the cells by specific JNK inhibitor causes a decrease of phosphorylated JNK expression and insensitivity of the cells to apoptosis. This result suggests an effect of DSF and Cu combination on the activation of the JNK signaling pathway [59].

The stem cells of multiple myeloma are considered to be one of the causes leading to the relapse of the disease, and their eradication can be an effective strategy for improving MM treatment. The characteristics of stem cells have been studied, such as the role of aldehyde dehydrogenase (ALDH) enzyme, isolated from MM, while evaluating the effect of DSF with Cu on the MM cells in vitro and in vivo. The exact mechanism of inhibition of these cells was also studied. The results show that ALDH^+^ cells contribute to tumorigenesis in vitro and in vivo. As in other studies, an increased effect of DSF in combination with copper was observed. DSF with Cu decreases the activity of ALDH, disrupts the ability of the clonogenic cell survival, and in vivo inhibits tumor proliferation and decreases the expression of stem cell transcription factors. The results showed that DSF with Cu effectively inhibits MM in vitro as well as in vivo and has no effect on healthy hematopoietic stem cells. These findings support the potential therapeutic use of disulfiram in eradication of the population of myeloma stem-cell-like cells [60].

## 3. Conclusions

Promising treatment results after disulfiram application in different types of cancer have been described in several studies. This review supports the importance of repurposing disulfiram as a potential anticancer agent in scientific and follow-up clinical trials, focusing on multiple myeloma treatment. Because the new treatment options are targeted at patients with relapsed multiple myeloma, disulfiram appears to be effective in treating this disease stage. Due to its long-term use in the treatment of alcohol dependence, the disulfiram side effects are well known. In addition, its low price is also a significant factor. 

Despite the low number of studies performed on myeloma cell lines, the inhibitory effect of the disulfiram and copper combination on malignant plasma cell progression is evident. Because malignant plasma cells generate large amounts of defective paraprotein, it is advisable to aim the therapy at the degradation of proteins and apply the so far most efficient bortezomib, targeting the proteasome. Nevertheless, the effect of disulfiram is not negligible as it deactivates the signaling pathway of ubiquitin-proteasome degradation, the p97-NPL4-UFD1. Our current study supports the inclusion of disulfiram into experimental or clinical studies, with the aim of its therapeutic use in MM patients.

## Figures and Tables

**Table 1:** The CRAB criteria used in the diagnosis of multiple myeloma [8].

FEATURE	DIAGNOSTIC CRITERIA
C—Calcium	serum calcium >0.25 mmol/L (>1 mg/dL) higher than the upper limit of normal or >2.75 mmol/L (>11 mg/dL)
R—Renal Insufficienci	creatinine clearance <40 mL per minute or serum creatinine >177 mol/L (>2 mg/dL)
A—Anemia	hemoglobin valure of >20 g/L below the lowest limit of normal, or a hemoglobin value <100 g/L
B—Bone Disease	one or more osteolytic lesion on skeletal radiography, CT, or PET/CT
Source: International Myeloma Working Group (IMWG) Br. J. Haematol., 2003, 121:749–757	
